# Computing Techniques for Environmental Research and Public Health

**DOI:** 10.3390/ijerph18189851

**Published:** 2021-09-18

**Authors:** Gwanggil Jeon, Abdellah Chehri

**Affiliations:** 1 Department of Embedded Systems Engineering, College of Information Technology, Incheon National University, Incheon 22012, Korea; 2 Department of Applied Sciences, University of Quebec at Chicoutimi, Chicoutimi, QC G7H 2B1, Canada; achehri@uqac.ca

Human bodies are continuously generating information about our health. This information can be assessed by physiological tools that gauge bio-signals such as blood pressure, heart rate, and blood glucose. Computer scientists are obtaining knowledge on new technologies to manage these bio-signals using a range of equations and approaches. Currently, real-time monitoring, cloud computing, edge computing, and the Internet of Things are key technologies that can be applied in the environmental research and public health field.

In light of these and many other challenges, a Special Issue entitled *Computing Techniques for Environmental Research and Public Health* has been dedicated to address the current status, challenges, and future research priorities for environmental research and public health.

Starting from the above considerations, this Special Issue aims to investigate the impact of adopting advanced and innovative information computing-based algorithms in environmental research and public health field applications, including ones that take advantage of recent big data, compression, multichannel, sensor, and prediction techniques. This edition of the Special Issue is focused primarily on computing techniques for environmental research and public health applications with a particular emphasis on artificial intelligence and signal processing platforms. This issue is intended to provide a highly recognized international forum to present recent advances in the *International Journal of Environmental Research and Public Health.* We welcome both theoretical contributions as well as papers describing interesting applications. Papers were invited for this Special Issue considering aspects of this problem, including:

• public health;

• bio-signal processing;

• the Internet of Things;

• real-time monitoring;

• cloud computing.

After review, a total of seven papers have been accepted for publication in this issue.

Deep-learning (DL)-based methods are of growing importance in the field of single image super-resolution (SISR). However, the practical application of these DL-based models is an enduring problem due to the requirement of heavy computation and huge storage resources. Furthermore, the powerful feature maps of hidden layers in convolutional neural networks (CNN) help the model to learn useful information. However, there exists redundancy among feature maps, which can be further exploited. To address these issues, the contribution by Yu et al. [1], *Real-Time Environment Monitoring Using a Lightweight Image Super-Resolution Network*, proposes a lightweight efficient feature generating network (EFGN) for SISR by constructing the efficient feature generating block (EFGB). Specifically, the EFGB can conduct plain operations on the original features to produce more feature maps with slightly increased parameters. With the help of these extra feature maps, the network can extract more useful information from low resolution (LR) images to reconstruct desired high resolution (HR) images. Experiments conducted on the benchmark datasets demonstrate that the proposed EFGN can outperform other deep-learning based methods in most cases and possess relatively lower model complexity. Additionally, the running time measurement indicates the feasibility of real-time monitoring. 

In the contribution by Bouhassoune et al. [2], *Optimization of UHF RFID Five-Slotted Patch Tag Design Using PSO Algorithm for Biomedical Sensing Systems*, a new flexible, wearable radio frequency identification (RFID) five-shaped slot patch tag placed on the human arm is designed for ultra-high frequency (UHF) healthcare sensing applications. The compact proposed tag consists of a patch structure with five shaped slot radiators and a flexible substrate, which minimizes the human body’s impact on the antenna radiation performance. The authors have optimized their designed tag using the particle swarm optimization (PSO) method with curve fitting within MATLAB to minimize antenna parameters to achieve a good return loss and an attractive radiation performance in the operating band. The PSO-optimized tag’s performance has been examined over the specific placement in some parts of the human body, such as the wrist and chest, to evaluate the tag response and enable their tag antenna conception in wearable biomedical sensing applications. Finally, they have tested the robustness of this tag by evaluating its sensitivity as a function of the antenna radiator placement over the ground plane or by shaping the ground plane substrate for the tag’s position from the human body. Their numerical results show an optimal tag size with good matching features and promising read ranges near the human body. 

The global outbreak of the COVID-19 pandemic has uncovered the fragility of healthcare and public health preparedness and planning against epidemics/pandemics. In addition to the medical practice for treatment and immunization, it is vital to have a thorough understanding of community spread phenomena as related research reports that 17.9–30.8% of confirmed cases remained asymptomatic. Therefore, an effective assessment strategy is vital to maximizing the tested population in a short amount of time. The contribution by Simsek et al. [3], *Artificial Intelligence-Empowered Mobilization of Assessments in COVID-19-like Pandemics: A Case Study for Early Flattening of the Curve*, proposes an artificial intelligence (AI)-driven mobilization strategy for mobile assessment agents for epidemics/pandemics. To this end, a self-organizing feature map (SOFM) is trained using data acquired from past mobile crowdsensing (MCS) campaigns to model mobility patterns of individuals in multiple districts of a city to maximize the assessed population with minimum agents in the shortest possible time. Through simulation results for a real street map on a mobile crowdsensing simulator and considering the worst-case analysis, the risk of community spread on the 15th day following the first confirmed case in the city is shown. An AI-enabled mobilization of assessment centers can reduce the unassessed population size to one-fourth of the unassessed population under the case when assessment agents are randomly deployed over the entire city.

Hearing loss is a disease exhibiting a growing trend due to a number of factors, including but not limited to the mundane exposure to noise and ever-increasing size of the older population. In the framework of a public health policymaking process, modeling the hearing loss disease based on data is a key factor in alleviating the issues related to the disease and in issuing effective public health policies. The contribution by Brdarić et al. [4], *A Data-informed Public Health Policy-Maker Platform*, studies the public health policymaking process. First, the paper describes the steps of the data-driven policymaking process. Afterward, a scenario along with the part of the proposed platform responsible for supporting policymaking is presented. With the aim of demonstrating the capabilities and usability of the platform for the policy-makers, some initial results of preliminary analytics are presented in the framework of a policy-making process. Ultimately, the utility of the approach is validated throughout the results of the survey, which was presented to health system policy-makers involved in the policy development process in Croatia. 

Algorithms for measuring semantic similarity between gene ontology (GO) terms have become a popular area of research in bioinformatics as they can help to detect functional associations between genes and potential impact on the health and wellbeing of humans, animals, and plants. While the focus of the research is on the design and improvement of GO semantic similarity algorithms, there is still a need to implement such algorithms before they can be used to solve actual biological problems. This can be challenging given that the potential users usually come from a biology background and are not programmers. A number of implementations exist for some well-established algorithms, but they are not generic enough to support any algorithm other than the ones they are designed for. The aim of the contribution by Tsaramirsis et al. [5], *More Agility to Semantic Similarities Algorithm Implementations*, is to shift the focus away from implementation, allowing researchers to focus on algorithm design and execution rather than implementation. This is achieved by an implementation approach capable of understanding and executing user-defined GO semantic similarity algorithms. Questions and answers were used for the definition of the user-defined algorithm. Additionally, this approach understands any direct acyclic digraph in an open biomedical ontologies (OBO)-like format and its annotations. On the other hand, software developers of similar applications can also benefit by using this as a template for their applications. 

With the remarkable improvement in people’s socioeconomic living standards worldwide, adolescent obesity has increasingly become an important public health issue that cannot be ignored. Thus, in the contribution by Kim et al. [6], *Predicting Factors Affecting Adolescent Obesity Using General Bayesian Network and What-If Analysis*, the authors have implemented these techniques in an attempt to explore the viability of scenario-based simulations through the use of a data mining approach. In doing so, they wanted to examine the merits of using a general Bayesian network (GBN) with what-if analysis while exploring how it can be utilized in other areas of public health. The authors analyzed data from the 2017 Korean Youth Health Behavior Survey conducted directly by the Korea Centers for Disease Control and Prevention, including 19 attributes and 11,206 individual data points. Their simulations found that manipulating the amount of pocket money—between $60 and $80—coupled with a low-income background has a high potential to increase obesity compared with other simulated factors.

Additionally, when they manipulated an increase in studying time with a mediocre academic performance, it was found to potentially increase pressure on adolescents, which subsequently led to an increased obesity outcome. Lastly, they found that when they manipulated an increase in a father’s education level while manipulating a decrease in a mother’s education level, this had a large effect on the potential adolescent obesity level. Although obesity was the chosen case, this paper acts more as a proof of concept in analyzing public health through GBN and what-if analysis. Therefore, it aims to guide health professionals into potentially expanding their ability to simulate certain outcomes based on predicted changes in certain factors concerning future public health issues. 

Globally, water scarcity has become a common challenge across many regions. Digital surveillance holds promise for monitoring environmental threats to population health due to severe drought. India’s 2019 Chennai water crisis resulted in severe disruptions to social order and daily life, with local residents suffering due to water shortages. The contribution by Xiong et al. [7], *Digital Surveillance for Monitoring Environmental Health Threats: A Case Study Capturing Public Opinion from Twitter about the 2019 Chennai Water Crisis* explored public opinion captured through the Twitter social media platform and whether this information could help local governments with their emergency response. Sentiment analysis and topic modeling were used to explore public opinion through Twitter during the 2019 Chennai water crisis. The latent Dirichlet allocation (LDA) method identified topics that were most frequently discussed. A naïve Tweet classification method was built, and Twitter posts (called tweets) were allocated to identified topics. Topics were ranked, and corresponding emotions were calculated. A cross-correlation was performed to examine the relationship between online posts about the water crisis and actual rainfall, determined by precipitation levels. During the Chennai water crisis, Twitter users posted content that appeared to show anxiety about the drought’s impact and expressed concerns about the government’s response. Twitter users also mentioned causes for the drought and potential sustainable solutions, which appeared to be mainly positive in tone. Discussions on Twitter can reflect popular public opinion related to emerging environmental health threats. Twitter posts appear viable for informing crisis management as real-time data can be collected and analyzed. Governments and public health officials should adjust their policies and public communication by leveraging online data sources to inform disaster prevention measures.

The articles presented in this Special Issue provide insights into fields related to computing techniques for environmental research and public health, including models, performance evaluations and improvements, and application developments. We hope the readers can benefit from the insights of these papers and contribute to these rapidly growing areas. We also hope that this Special Issue will shed some light on major developments in areas of interest for the *International Journal of Environmental Research and Public Health* and attract the attention of the scientific community to pursue further investigation, leading to the rapid implementation of these technologies.
